# Element accumulation patterns and bioindicator potential of *Soldanella carpatica* along a montane–alpine gradient

**DOI:** 10.1007/s10661-026-15471-2

**Published:** 2026-05-22

**Authors:** Zuzana Kompišová Ballová, Gabriela Zatkalíková, Marián Janiga

**Affiliations:** Institute of High Mountain Biology, Žilina University, Tatranská Javorina 7, 05956 Tatranská Javorina, Slovak Republic

**Keywords:** Carpathian snowbell, ED-XRF spectroscopy, Altitudinal gradient, Atmospheric deposition, Trace elements, Elemental interactions

## Abstract

This study analyzed the multielement composition of *Soldanella carpatica* and the surrounding soils along a montane–alpine gradient (1137–1861 m a.s.l.) across seven sampling sites in the Tatra Mountains (Western Carpathians, Slovakia) to understand how altitude with associated habitat conditions, seasonality, and soil chemistry influence element accumulation and distribution patterns. The aim was to explore multielement relationships and possible environmental drivers affecting the uptake and distribution of selected elements within plant organs (roots, stems, and leaves) and surrounding soils. Portable energy-dispersive X-ray fluorescence was used to quantify biogenic (S, Cl, K, Ca, and Mn) and trace elements (Sr, Ba, Zn, and Pb). Total mercury was analyzed separately using a direct mercury analyzer (DMA-80), with mean concentrations around 0.02 ± 0.01 mg kg⁻^1^ (dry weight) in plant tissues and 0.14 ± 0.08 mg kg⁻^1^ (dry weight) in soils. Eighty-nine plant samples and associated soil samples were analyzed along the gradient. Biogenic elements (S, Cl, K, and Ca) were most abundant in aboveground tissues, indicating efficient uptake and translocation. In contrast, potentially toxic trace elements (Hg, Pb, Zn, Sr, and Ba) primarily accumulated in roots and rhizospheric soil, suggesting limited mobility and protective retention mechanisms. Elemental patterns varied with vegetation type and altitude: open meadows showed higher Cl, K, Ca, and Ba concentrations, consistent with greater exposure to atmospheric inputs, while forested sites exhibited Mn enrichment linked to acidic soils and canopy throughfall. Principal component analysis explained approximately 85% of the total variance, revealing an antagonistic Mn–Sr relationship and a shared loading pattern of Zn and Pb. These patterns may partly reflect contrasting geochemical behaviour and possible atmospheric influences rather than direct source attribution. The element-specific allocation patterns of *S. carpatica* highlight its potential as a sensitive bioindicator of element accumulation in high-elevation ecosystems.

**Supplementary Information:**

The online version contains supplementary material available at 10.1007/s10661-026-15471-2.

## Introduction

The occurrence of certain high-mountain plant species can provide valuable insight into environmental pollution (Zaghloul et al., 2020), as mountain ranges often function as long-term accumulation zones for atmospherically transported contaminants (Jiao et al., 2021). Vascular plants inhabiting high elevations are exposed to a combination of extreme environmental constraints, including low temperatures, intense solar radiation, and short growing seasons. At the same time, high-mountain plants must cope with elevated concentrations of potentially toxic elements, which may exert stronger stress effects in alpine environments than at lower elevations (Ghori et al., 2019). Such stress responses are often supported by symbiotic associations with mycorrhizal fungi. These associations enhance nutrient acquisition and increase tolerance to metal stress (Bano & Ashfaq, 2013; Zubek et al., 2009). In this study, we examine the concentrations and interactions of chemical elements in the Carpathian snowbell (*Soldanella carpatica*), a species endemic to the Carpathians and parts of the Eastern Balkans. *S. carpatica* is well adapted to montane, alpine, and subnival environments and exhibits pronounced phenotypic variability along elevational gradients, making it a suitable model species for investigating environmental controls on elemental uptake and allocation (Kiełtyk, 2024). The species typically occurs between 1000 and 2500 m a.s.l. It inhabits well-drained, slightly acidic soils rich in organic matter and experiences cool temperatures, high precipitation, and prolonged winter snow cover (Kiełtyk, 2024; Valachovič et al., 2019). From a functional perspective, it is a hemicryptophyte with a stress-tolerant life strategy, allocating a substantial proportion of its biomass to belowground storage organs (Grime, 2001).

Plants take up both essential nutrients and potentially toxic elements (PTEs) from their environment (Kumar et al., 2021; Thalassinos et al., 2023). While the majority of elements enter plant tissues via the root system, foliar uptake from atmospheric deposition can also contribute to elemental composition, particularly in high-elevation ecosystems where cloud water and fog deposition are frequent (Rao, 2009). Therefore, element accumulation reflects both soil-derived and atmospheric inputs, as well as their interactions within plant tissues.

Interactions among elements (synergistic or antagonistic) can influence uptake, transport, and physiological functioning (Bamagoos et al., 2022; Ni et al., 2024; Rietra et al., 2017). Although such interactions are well documented in agricultural crops (e.g., Aboyeji et al., 2020; Prasad et al., 2016; Rietra et al., 2017), they remain poorly understood in wild-growing plants, particularly in alpine and montane ecosystems exposed to long-range atmospheric deposition.

High-mountain regions are widely recognized as sensitive indicators of environmental change because they integrate the effects of climatic variability and atmospheric pollutant deposition over long time scales. The Tatras are characterized by low mean temperatures, high annual precipitation (approximately 1200–2130 mm), frequent temperature inversions, and regular formation of mountain fog (Konček et al., 1973; Tarábek, 1980). Previous studies have identified the Tatras, as part of the Western Carpathians, among the more polluted mountain regions in Europe, influenced by long-range atmospheric transport of pollutants from both regional and distant industrial sources (Ballová & Janiga, 2018; Haas et al., 2025). These conditions make the Tatras a valuable natural laboratory for studying element accumulation and distribution in relatively remote environments.

Recent research increasingly applies multielement analytical approaches to alpine and montane vegetation, enabling more comprehensive assessments of nutrient accumulation patterns and interactions with potentially toxic elements under natural conditions. Elemental concentrations were determined using energy-dispersive X-ray fluorescence (ED-XRF), a well-established technique widely applied in environmental studies. ED-XRF enables rapid, multielement screening of heterogeneous soil and plant samples with minimal sample preparation and detection limits suitable for ecological applications (Okkenhaug et al., 2023). It also shows strong agreement with reference laboratory methods in environmental risk assessments (Liu et al., 2022). Compared to laser-induced breakdown spectroscopy (LIBS), which can achieve low detection limits (Kumar et al., 2023) but is more sensitive to matrix effects and requires complex calibration (Nunes et al., 2022), ED-XRF is generally more robust for routine screening of natural plant and soil matrices.

Building on this approach, the primary aim of the present study was to investigate organ-specific patterns of elemental accumulation and interaction in *S. carpatica* along a montane-to-alpine gradient. Specifically, we examined how altitude, seasonality, and soil chemistry influence the uptake and internal distribution of essential nutrients and selected potentially toxic elements in this endemic high-mountain species. By integrating plant- and soil-based data across elevation zones, this study evaluates the suitability of *S. carpatica* as a bioindicator of integrated environmental inputs in alpine and montane ecosystems, with emphasis on multielement interactions rather than single-element accumulation patterns.

## Materials and methods

### Field sampling

**Fig. 1:**
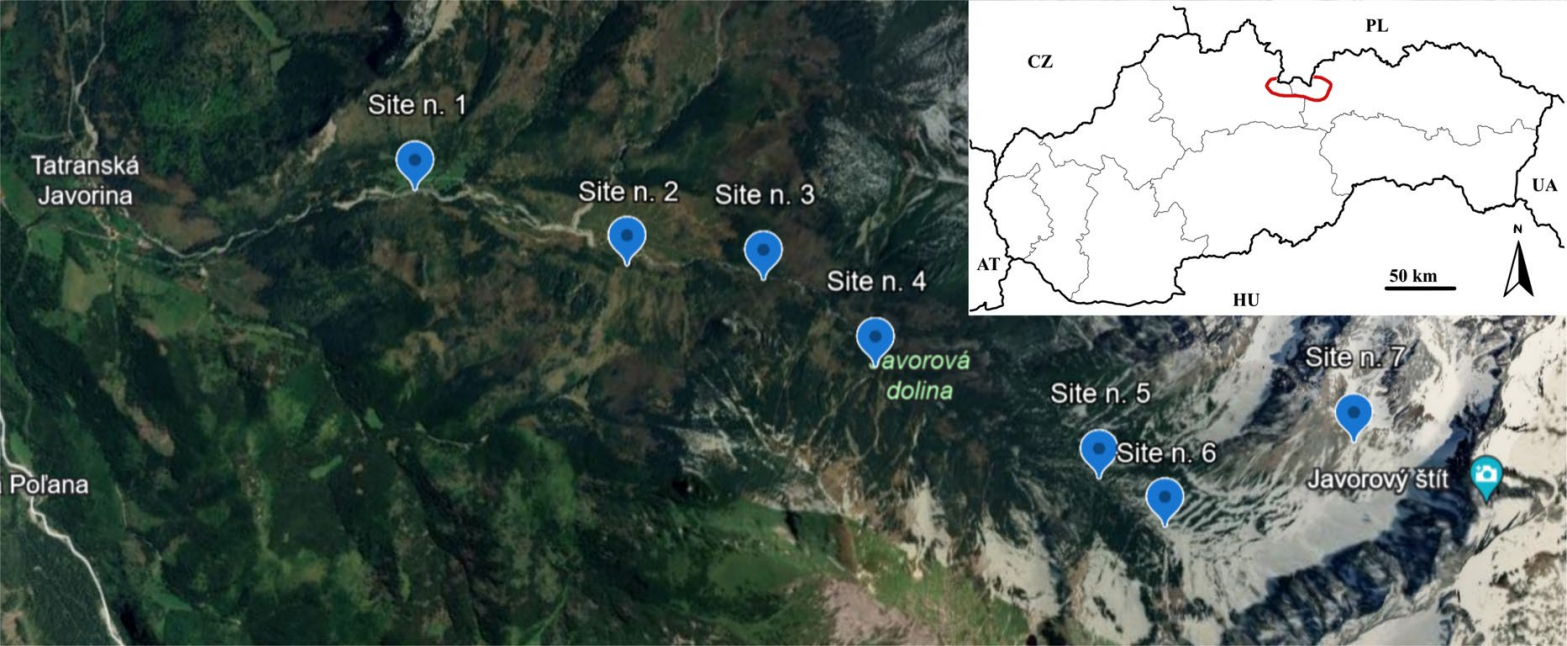
Sampling sites along the elevational transect in Javorová dolina (High Tatras) and the location of the Tatra Mountains within Slovakia: site 1–1137 m a.s.l.; site 2–1213 m a.s.l.; site 3–1270 m a.s.l.; site 4–1390 m a.s.l.; site 5–1470 m a.s.l.; site 6–1554 m a.s.l.; and site 7–1878 m a.s.l. The Tatra Mountains, including Javorová dolina, are highlighted in red on the map (source: Google Earth). Sampling positions varied within each site but remained within a 30-m radius of the indicated locations

During October 2021–September 2023, *S. carpatica* and surrounding soil were sampled along a vertical transect in Javorová dolina (High Tatras) at seven sites distributed at approximately 100-m elevation intervals (1137, 1213, 1270, 1390, 1470, 1554, and 1878 m a.s.l.; Fig. 1), extending from the valley floor to the tarn Žabie Javorové pleso. Sites 2–4 (1213–1391 m a.s.l.) were located under a closed spruce canopy, whereas the open sites included site 1 (1137 m a.s.l., forest edge), site 5 (1470 m a.s.l., upper forest boundary at the transition to the subalpine zone), the subalpine site 6 (1554 m a.s.l.), and the alpine site 7 (1878 m a.s.l.). Sampling locations slightly varied within each site according to plant occurrence but remained within a 30-m radius of the original position. Whole plants were excavated and separated into roots, stems and leaves; flowers were excluded from statistical analyses due to uneven seasonal occurrence. Each sampling campaign involved new individuals, i.e. repeated sampling was not conducted on the same plants. The number of samples per elevation site, seasonal period and organ is summarized in Supplementary Table S1.

Monthly sampling was conducted at all elevations from June to November, covering the main vegetation period. Sampling in May was limited to the two lowest sites, whereas winter and early-spring sampling (December–April) was restricted to the lowest site and partly to the second lowest site due to snow cover and limited accessibility at higher elevations (Supplementary Table S2).

At each sampling site, surface soil samples were collected to a depth of 2 cm after removing surface vegetation and litter; the average mass of each composite soil sample was approximately 150 g. During spring sampling, plant material comprised both new shoots and individuals that had overwintered beneath the snow cover, reflecting the phenological heterogeneity of *S. carpatica* at the beginning of the growing season.

### Laboratory analyses

#### Sample preparation

Plant samples were thoroughly washed with distilled water to remove impurities and contamination with elements from atmospheric deposition or soil. Special attention was paid to roots, which were carefully washed to remove adhering soil particles. Soil samples were air-dried at room temperature, gently disaggregated by hand, and coarse fragments (stones, roots, and visible organic debris) were removed prior to milling. To prevent potential losses of mercury due to volatilization, plant and soil samples were dried separately at room temperature for 14 days rather than in a drying oven. After drying, the samples were ground to powder in a cryogenic ball mill (RETSCH Cryomill, Germany). The grinding container was washed with distilled water after each sample to avoid contamination. The grinding frequency was set at 30 Hz for each sample. Grinding time was 40 s to 2 min for plant samples, while soils required 2 to 4 min for complete homogenization and were subsequently sieved through a 0.071-mm mesh.

#### Elemental analysis using ED-XRF

Elemental composition of plant and soil samples was analyzed by energy-dispersive X-ray fluorescence (ED-XRF; Delta, BAS Innov-X, USA), a rapid multielement screening technique widely applied in environmental studies (Marguí et al., 2022). Although the instrument is primarily designed for mineralogical and metallurgical applications, ED-XRF has been successfully adapted for environmental matrices, including soils and plant tissues, providing reproducible semiquantitative data suitable for comparative ecological analyses (da Costa et al., 2023; Montanha et al., 2020; Ribeiro et al., 2021).

Each homogenized and ground sample was placed into a plastic sample cup with a thin transparent prolene film at the base. Samples were packed uniformly to ensure full and even coverage of the film surface, and the powder layer was standardized to approximately 1 cm thickness. Instrument calibration was performed using a CONOSTAN standard, and matrix-specific standardization was achieved by measuring certified reference materials: INCT-PVTL-6 Polish Virginia Tobacco Leaves (ICHTJ, Poland) for plant samples and SRM 2711a Montana II Soil (NIST, USA) for soil samples. For each element, calibration factors were derived from three replicate CRM measurements and applied to correct sample concentrations. Each sample was measured using three excitation beams (80 s per beam), repeated three times, and averaged to obtain final values.

#### Quality assurance and quality control (QA/QC)

Analytical accuracy was independently verified using certified reference materials: INCT-PVTL-6 Polish Virginia Tobacco Leaves (ICHTJ, Poland) and SRM 1575a Pine Needles (NIST, USA) for plant matrices, and SRM 2711a Montana II Soil (NIST, USA) and ERM-CC141 Loam Soil (IRMM, Belgium) for soil matrices. All CRMs were analyzed in triplicate. Recoveries for the quantified elements (S, Cl, K, Ca, Mn, Zn, Rb, Sr, Ba, and Pb) were within acceptable ranges for ED-XRF screening of environmental matrices (typically ~ 90–105%), and relative standard deviations (RSD) were below 10%, indicating satisfactory analytical accuracy and repeatability Supplementary Table S3.

Instrument performance and measurement consistency were monitored by reanalysis of reference materials after every ten samples. Procedural blanks were analyzed in triplicate after every ten measurements. For plant-type matrices, laboratory starch and analytical-grade agarose were used as organic blank materials, whereas high-purity SiO_₂_ powder served as the blank matrix for soil analyses. In addition, granulated laboratory paraffin was measured periodically as an inert material to verify instrumental background stability and potential contamination of the measurement window.

Limits of detection (LOD) were derived from instrument-reported values generated by the ED-XRF software using Compton normalization to account for matrix effects (Innov-X Systems, 2010). When an element concentration was reported by the instrument as < LOD, the corresponding value represents the instrument detection limit defined as three times the standard deviation of the background signal $(\mathrm{LOD}=3\upsigma)$. For elements that were consistently detected in blank measurements, the analytical uncertainty reported by the instrument (± = 1σ) was used to estimate conservative proxy detection limits according to $\mathrm{LOD}=3\times\upsigma$ and $\mathrm{LOQ}=10\times\upsigma$. Matrix-dependent LOD and LOQ values derived from blank measurements are summarized in Supplementary Table S4. Because portable ED-XRF provides matrix-dependent screening-level detection limits, the reported LOD values represent practical analytical thresholds under the applied measurement conditions rather than absolute instrumental detection limits.

#### Mercury analysis (Hg)

Mercury concentrations in plant and soil samples were determined using a Milestone DMA-80 Direct Mercury Analyzer (Milestone, Italy). Sample masses ranged from 0.085 to 0.15 g and were recorded using a precision balance (KERN 770, Kern & Sohn, Germany). Results were expressed on a dry-weight basis. Samples were analyzed in nickel boats, which were thoroughly cleaned and thermally decontaminated between measurements to prevent carry-over. Mercury was released by thermal decomposition and quantified by atomic absorption spectrometry, following the manufacturer’s standard operating procedure.

#### Quality assurance and quality control (QA/QC) for Hg

Analytical accuracy was validated using certified reference materials (CRMs): INCT-PVTL-6 Polish Virginia Tobacco Leaves (ICHTJ, Poland) and SRM 1575a Pine Needles (NIST, USA) for plant matrices, and SRM 2711a Montana II Soil (NIST, USA) for soil matrices. All CRMs were analyzed in triplicate at the beginning and end of each analytical run. Recoveries ranged between 95 and 105% across the CRMs, and precision was high (RSD < 5%), indicating excellent repeatability.  

Procedural blanks (empty, pre-cleaned nickel boats) were analyzed in triplicate after every ten samples to monitor baseline stability. Limits of detection (LOD) and quantification (LOQ) were calculated from ten independent blank measurements performed at the start of the analysis $(\mathrm{LOD}=3\times \mathrm{SD}\_ \mathrm{blank};\;\mathrm{LOQ}=10\times \mathrm{SD}\_ \mathrm{blank})$. Based on these measurements and an average sample mass of 0.1 g, the resulting LOD and LOQ were 0.003 µg g⁻^1^ and 0.009 µg g⁻^1^, respectively. These values were well above the absolute instrumental detection limit (0.003 ng Hg per measurement).

### Statistical analyses

**Fig. 2:**
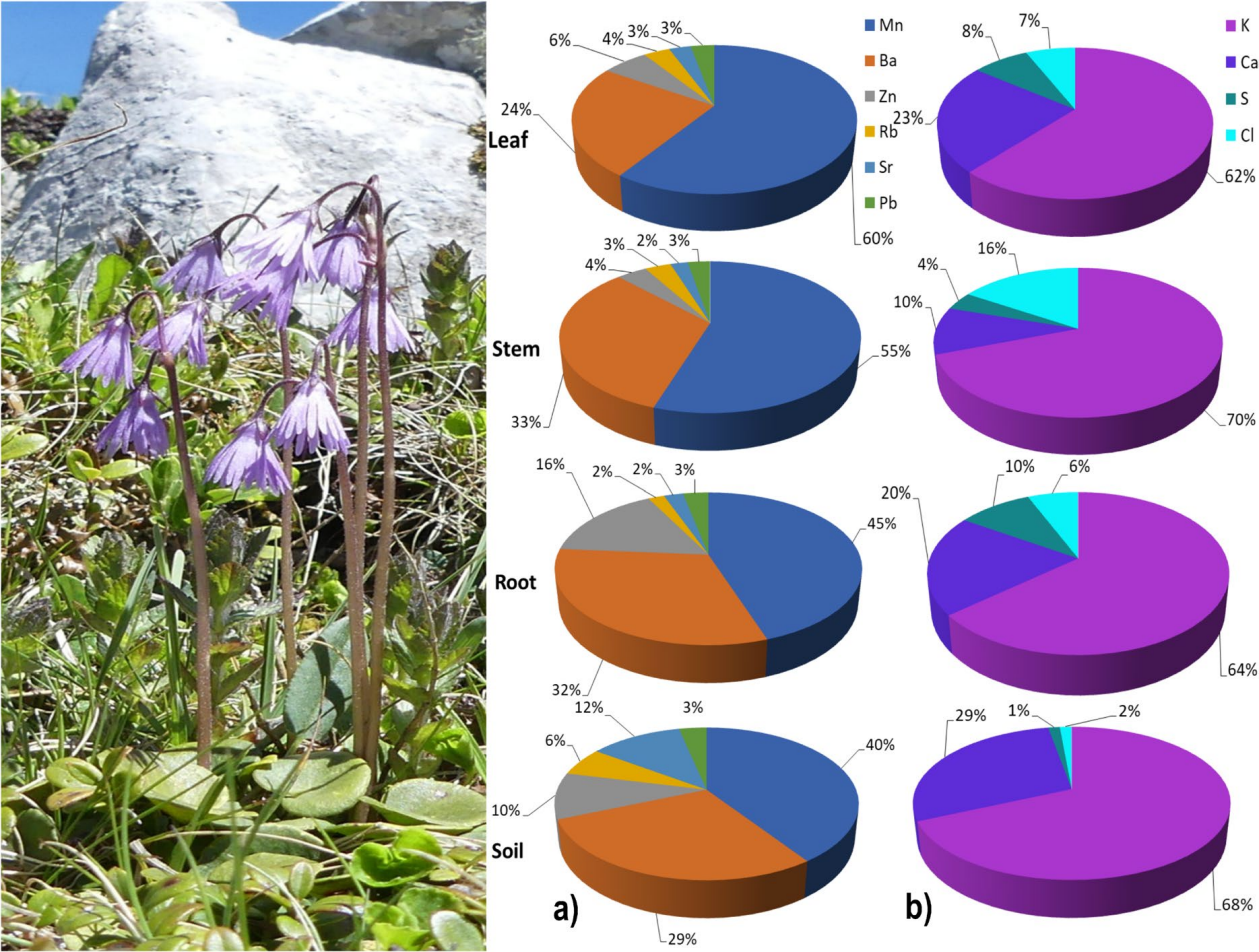
Percentage accumulation of elements in soil and plant organs of *S. carpatica* (foto: Zuzana Kompišová Ballová) according to their mean detected amounts: (a) elements with lower percentage concentrations and (b) elements with higher percentage concentrations. Only 10 elements that were successfully measured and calibrated using ED-XRF spectroscopy are included; other elements present in the plant or soil are not shown. Values represent the relative contribution of each element to the total of the measured elements. Mercury (Hg) is not displayed because its relative concentration was negligible and appeared as 0% compared to the other elements

Only elements measured above the detection limit and reliably calibrated (S, Cl, K, Ca, Mn, Zn, Hg, Rb, Sr, Ba, and Pb) were included in the analyses. Descriptive statistics were calculated for plant organs and soil, and relative element distributions were visualized (Fig. 2a, and b). The elements in the graph were divided into two groups to make the graph easier to read, namely, elements with lower mean concentrations and elements with higher mean concentrations. The bioconcentration factor (BCF) was used to assess element uptake from soil (Tong et al., 2022):

$$BCF\hspace{0.17em}=\hspace{0.17em}C_{root}/C_{soil},\;BCF=C_{stem}/C_{soil},\;BCF=C_{leaf}/C_{soil}$$

where *C*_*root*_, *C*_*stem*_, and *C*_*leaf*_ denoted element concentrations in roots, stems, and leaves, and *C*_*soil*_ was the concentration of elements in soil. A value of BCF ≤ 1 indicates that the plant can absorb the element but cannot accumulate it. Conversely, a value of BCF > 1 signifies that the plant possesses the ability to accumulate the element.

The translocation factor (TF) is defined as the ratio of total element concentration in aboveground plant parts to total element concentration in roots (Tong et al., 2022), and was calculated by the equations:

$$TF\hspace{0.17em}=\hspace{0.17em}C_{stem}/C_{root},\;TF=C_{leaf}/C_{root}$$

where *C*_*stem*_ and *C*_*leaf*_ were the concentrations of elements in stems and leaves, and *C*_*root*_ was the concentration of elements in roots. *TF* ≤ 1 is indicative of the low translocation capacity of elements from the root to stem or leaf tissues. Conversely, *TF* > 1 has indicated that the plant is able to translocate elements effectively from the root to stem or leaf tissues.

As ED-XRF provides screening-level concentration data, we focused on relative patterns and category-level trends rather than absolute values. Principal component analysis (PCA) was used to explore multi-element co-variation and synergistic relationships. PCA reduces the dimensionality of multivariate datasets by transforming correlated variables into a smaller set of uncorrelated components that retain most of the variance (Jolliffe & Cadima, 2016). Analyses were performed in STATISTICA 10 (StatSoft, Inc., 2011) using 11 elements with sufficient data (S, Cl, K, Ca, Mn, Zn, Hg, Rb, Sr, Ba, and Pb); loadings ≥|0.5| were considered strong. Sample sizes and mean concentrations of the measured elements for each plant organ and the soil matrix are provided in Tables 1 and 2; cases with missing values were excluded from further analyses. The distribution of samples across altitude levels, plant organs and sampling periods, which constitutes the dataset used for the PCA and subsequent statistical analyses, is summarized in Supplementary Tables S1 and S2.

**Table 1:** Mean concentrations (± SD; *N* = number of samples) of elements (in mg/kg dry wt.) in the leaf and stem of *S. carpatica* from the Javorová Valley (High Tatras 2021–2023)

Leaf				Stem		
	Mean	Std. Dev	*N*	Mean	Std. Dev	*N*
S	4291	2505	89	5313	2929	89
Cl	3767	3677	89	21,607	15,005	89
K	33,421	19,232	89	92,638	47,946	89
Ca	12,684	6620	89	13,656	7673	89
Mn	319	290	89	428	379	89
Zn	33.0	13.2	89	30.4	17.0	89
Hg	0.02	0.01	89	0.02	0.01	90
Rb	18.9	10.1	89	26.0	15.8	88
Sr	15.7	15	89	17.4	14.6	88
Ba	129	87	89	253	135	88
Pb	16.9	9.7	88	22.4	19.9	88

**Table 2:** Mean concentrations (± SD; *N* = number of samples) of elements (in mg/kg dry wt.) in the root of *S. carpatica* and surrounding soil from the Javorová Valley (High Tatras 2021–2023)

Root				Soil		
	Mean	Std. Dev	*N*	Mean	Std. Dev	*N*
S	4280	2112	87	451	281	89
Cl	2921	1,952	87	463	291	89
K	28,762	15,161	87	21,844	11,013	89
Ca	9005	5787	87	9190	8389	89
Mn	486	351	87	696	505	89
Zn	174.7	106.7	87	169.7	163.6	89
Hg	0.03	0.02	88	0.14	0.08	89
Rb	21.9	8.8	87	105.5	46.8	89
Sr	26.4	16.6	87	201.9	116.4	89
Ba	341	223	87	492	109	89
Pb	34.6	24.1	87	60.6	45.3	89

Data did not follow a normal distribution according to the Shapiro–Wilk test. Variances were heterogeneous for altitude and sample type (plant organ/soil) based on Levene’s test (*p* < 0.05); therefore, the effects of these factors on PCA scores were evaluated using Welch’s ANOVA implemented in PAST (Hammer et al., 2001), which is appropriate when variance homogeneity is violated and sample sizes are unequal (Zaiontz, 2024). Because several mid-elevation sites were located under a closed spruce canopy, canopy effects were evaluated together with altitude and were not treated as a separate factor. Welch’s ANOVA is robust to heteroscedasticity, unbalanced designs and nonnormal data, thereby reducing the risk of Type I errors (Martinez & Kudapa, 2024). Where Welch’s ANOVA indicated significant overall effects, post hoc differences among groups were evaluated using Tukey’s HSD test.

For seasonal effects, variance homogeneity was met for PC1–PC4 (Levene’s test, *p* > 0.05), and these components were assessed using one-way ANOVA of PCA scores, followed by Tukey’s HSD post hoc test for pairwise seasonal comparisons. In contrast, variance heterogeneity was detected for PC5 (Levene’s test, *p* < 0.05), and seasonal differences for this component were evaluated using Welch’s ANOVA. Due to sparse and uneven winter and spring sampling caused by persistent snow cover, data from December to May were excluded from seasonal analyses Supplementary Table S2. Mean concentrations presented in Tables 1 and 2 are provided as descriptive summaries of the overall dataset across plant organs and soil and were not used for testing differences among altitude levels or sampling periods.

## Results

### Element concentrations in plant organs and soil

The mean concentrations of the analyzed elements, divided into two categories—biogenic (S, Cl, K, Ca, Mn, and Zn) and potentially toxic trace elements (Hg, Rb, Sr, Ba, and Pb)—are presented in Table 1 for leaf and stem samples and in Table 2 for root and soil samples as descriptive statistics summarizing the overall dataset. The standard deviations and number of samples analyzed are also included.

### Percentage accumulation of elements in soil and plant organs

The relative distribution of element concentrations among plant organs and soil was presented in Fig. 2a and b. These values were expressed as percentages and should be regarded as relative, since not all elements present in *S. carpatica* were visualized. For clarity, the elements were divided into two groups based on their relative proportions in plant organs and soil. Elements with higher percentages included K, Ca, S, and Cl (Fig. 2b), whereas elements with lower percentages comprised Mn, Ba, Zn, Rb, Sr, and Pb (Fig. 2a). The average content of K followed the order: stem > soil > root > leaf. For Ca, the order was: soil > leaf > root > stem. Cl showed the pattern: stem > leaf > root > soil. Mn accumulated predominantly in the leaf, with the sequence: leaf > stem > root > soil. Sulfur was most abundant in the root, decreasing in the order: root > leaf > stem > soil. Zn accumulated mainly in the root, with the distribution: root > soil > leaf > stem. Among the potentially toxic trace elements, Rb, Sr, and Pb were highest in soil, suggesting limited translocation into plant organs (Rb: soil > leaf > stem > root; Sr: soil > leaf > stem = root; and Pb: soil = root = leaf = stem). In contrast, Ba was most concentrated in the stem (stem > root > soil > leaf). Mercury occurred at consistently low relative proportions compared to other elements and was therefore not emphasized in the percentage-based comparison.

### Bioconcentration factor (BCF) and translocation factor (TF)

The bioconcentration factor (*BCF*) and the translocation factors (*TF*) for elements in the organs of *S. carpatica* and surrounding soil are presented in Table 3. Higher accumulation capacity was observed mainly for the biogenic elements S, Cl, K, and Ca. The *BCF* for leaves relative to soil (*C*_*leaf*_*/C*_*soil*_) followed the order: S > Cl > K > Ca, while for stems relative to soil (*C*_*stem*_*/C*_*soil*_) it decreased in the order: Cl > S > K > Ca, and for roots relative to soil (*C*_*root*_*/C*_*soil*_) the trend was: S > Cl > K > Ca. Effective translocation between leaves or stem and root was evident for Cl, K, and Ca, and between stem and root also for S and Rb. The translocation factor (TF) from root to leaf (*C*_*leaf*_*/C*_*root*_) followed the order: Ca > Cl > K, whereas TF from root to stem (*C*_*stem*_*/C*_*root*_) decreased in the order: Cl > K > Ca > S > Rb.

**Table 3:** The bioconcentration factors (*BCF*) and the translocation factors (*TF*) for elements in the organs of *S. carpatica* and surrounding soil from the Javorová Valley (High Tatras 2021–2023). Values higher than one are italicized; these values indicate that the plant was capable of efficiently accumulating or translocating the respective element

Element	BCF C_leaf_/C_soil_	BCF C_stem_/C_soil_	BCF C_root_/C_soil_	TF C_leaf_/C_root_	TF C_stem_/C_root_
S	*9.51*	*11.78*	*9.49*	1.00	*1.24*
Cl	*8.13*	*46.66*	*6.31*	*1.29*	*7.40*
K	*1.53*	*4.24*	*1.32*	*1.16*	*3.22*
Ca	*1.38*	*1.49*	0.98	*1.41*	*1.52*
Mn	0.45	0.62	0.70	0.65	0.88
Zn	0.19	0.18	*1.03*	0.19	0.17
Hg	0.14	0.14	0.21	0.67	0.67
Rb	0.18	0.25	0.21	0.86	*1.19*
Sr	0.08	0.09	0.13	0.59	0.66
Ba	0.26	0.51	0.69	0.38	0.74
Pb	0.28	0.37	0.57	0.49	0.65

### Effects of altitude, seasonality, plant organ, and soil on synergistic impacts of elements

Table 4 presents the principal components (PCs) that most strongly explained the behaviour of elements in the plant organs of *S. carpatica* and the surrounding soil. The variance explained by the first five components was expressed as a percentage, with a total cumulative variance of 85.04%. PC1 was characterized by strong positive loadings of essential elements (S, Cl, and K) and strong negative loadings of potentially toxic trace elements (Hg, Zn, Rb, Sr, and Pb), indicating contrasting multivariate co-variation patterns between these two groups. These two groups exhibited an antagonistic relationship: an increase in biogenic (essential) elements in a sample was accompanied by a decrease in potentially toxic trace elements, and vice versa. PC2 was driven mainly by Cl, K, Ca, and Ba. The biogenic elements Cl, K, and Ca showed a synergistic trend with the potential pollutant Ba. PC3 represented an antagonistic relationship between Mn and Sr. PC4 captured the simultaneous variation of Zn and Pb, which indicated that these two elements tended to increase or decrease together. PC5 was dominated by Ca, whose behavior was explained primarily by this component.

**Table 4:** Principal component loadings (coordinates of variables) and percentage of variance explained by each principal component describing the behaviour of chemical elements in *S. carpatica*. The most important coordinates of variables which compose the appropriate component are italicized

Element	PC1	PC2	PC3	PC4	PC5
**Hg**	−*0.78*	0.29	0.18	−0.24	−0.15
**S**	*0.78*	0.43	−0.05	−0.24	0.09
**Cl**	*0.67*	*0.56*	0.08	0.03	−0.36
**K**	*0.64*	*0.66*	−0.09	0.16	−0.27
**Ca**	0.41	*0.54*	0.31	−0.13	*0.54*
**Mn**	−0.35	0.46	−*0.68*	0.23	0.17
**Zn**	−*0.55*	0.14	−0.42	−*0.52*	0.11
**Rb**	−*0.75*	0.31	−0.12	0.38	−0.16
**Sr**	−*0.67*	0.14	*0.51*	0.29	0.09
**Ba**	−0.47	*0.69*	0.09	0.21	0.19
**Pb**	−*0.59*	0.41	0.21	−*0.50*	−0.25
Variance	38.65%	20.74%	10.13%	9.16%	6.36%

#### Altitude

**Fig. 3:**
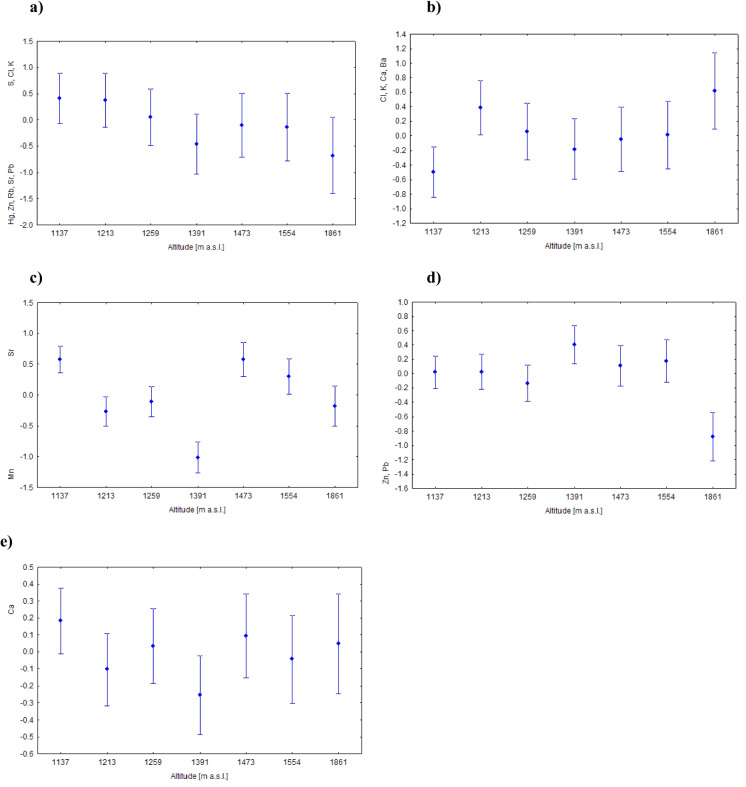
Means (± SE) of PCA scores for PC1–PC5 in relation to altitude (reflecting differences in site conditions): **a** PC1 (38.65% of explained variance) (Welch *F* = 1.804, df = 138.3, *p* > 0.05, n.s.); **b** PC2 (20.74%) (Welch *F* = 2.752, df = 140, *p* < 0.05); **c** PC3 (10.13%) (Welch *F* = 22.1, df = 134.2, *p* < 0.001); **d** PC4 (9.16%) (Welch *F* = 3.075, df = 153.3, *p* < 0.05); and **e** PC5 (6.36%) (Welch *F* = 2.358, df = 139.2, *p* < 0.05)

Because several mid-elevation sites were located under a closed spruce canopy (sites 2–4), altitudinal patterns should be interpreted as reflecting both elevation and associated site conditions. For PC1 (Fig. 3a), the antagonistic relationship between essential elements (S, Cl, and K) and potentially toxic trace elements (Hg, Zn, Rb, Sr, and Pb) did not vary significantly with sampling site or altitude. In contrast, PC2 (Fig. 3b) showed pronounced altitudinal effects: the concentrations of Cl, K, Ca, and Ba were lowest at 1137 m a.s.l. and highest at 1861 m a.s.l., with no clear differences among intermediate sites. PC3 revealed variation in the antagonistic relationship between Mn and Sr with the absence of a consistent altitudinal gradient (Fig. 3c). A simultaneous increase in Zn and Pb (PC4) was significant only at the highest site (1861 m a.s.l.; Fig. 3d). Finally, Ca (PC5) was identified as significantly different by Welch ANOVA (Fig. 3e), but no significant pairwise differences were detected among sites. Results of Tukey’s HSD test for PC2–PC5 are summarized in Table 5.

**Table 5:** Results of Tukey’s HSD test for differences among altitudes in factor scores (Factors 2–5). Different letters indicate statistically significant differences (*p* < 0.05)

Altitude (m a.s.l.)	Factor 2	Factor 3	Factor 4	Factor 5
1137	a	a	a	a
1213	b	b	a	a
1259	a,b	b	a	a
1391	a,b	c	a	a
1473	a,b	a,b	a	a
1554	a,b	b	a	a
1861	b	b,c	b	a

Different letters indicate significantly different groups (p < 0.05). Altitudes sharing the same letter are not significantly different. Altitudes with combined letters (e.g., 1473 = a,b) do not belong clearly to a single group and share similarity with multiple groups

#### Seasonality

**Fig. 4:**
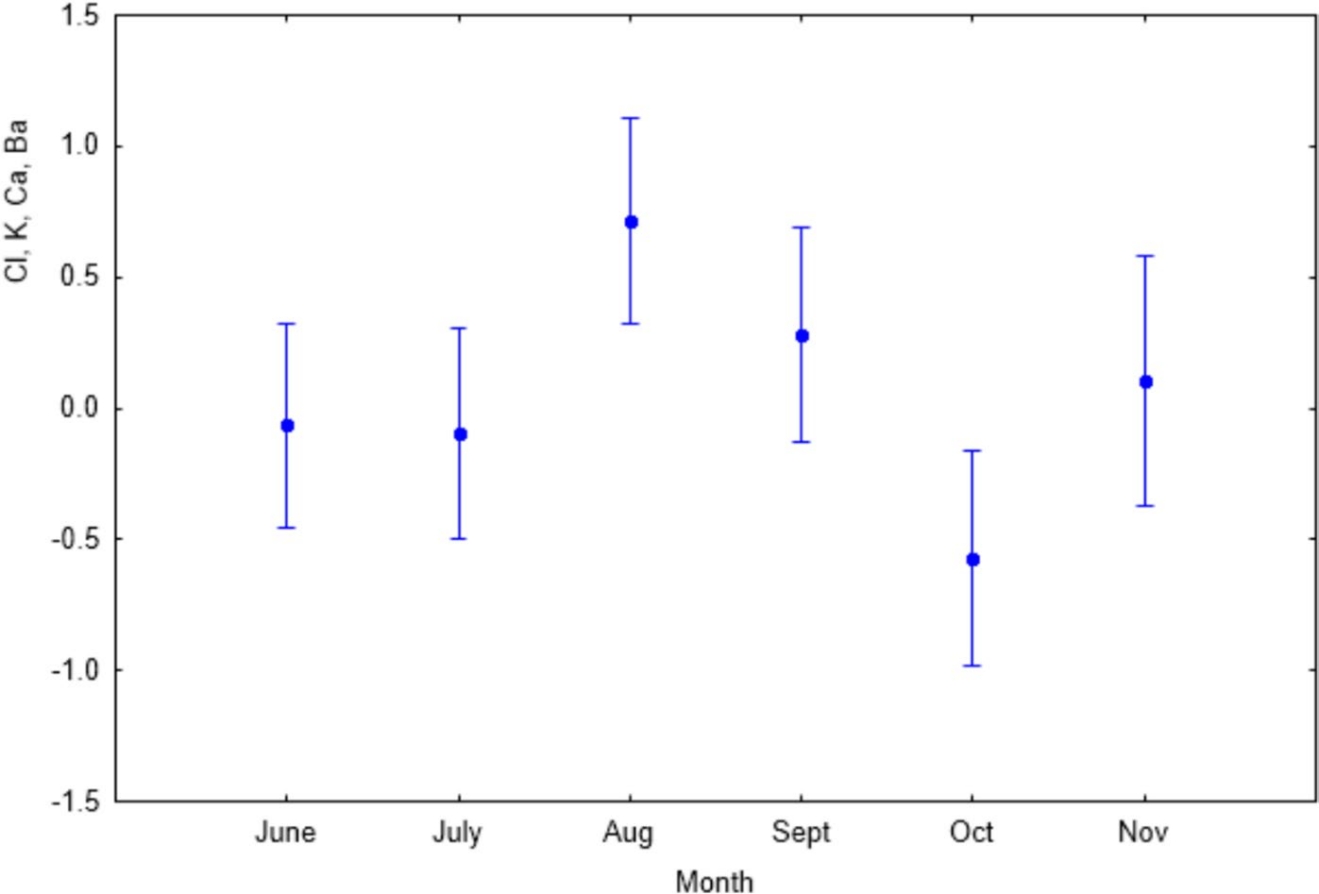
Means (± SE) of PCA scores for PC2 (20.74% of explained variance) in relation to seasonality (one-way ANOVA, *F*(5, 293) = 4.42, *p* < 0.001)

The antagonistic relationship described by PC1 (S, Cl, K vs. Hg, Zn, Rb, Sr, and Pb) was not significantly affected by season (one-way ANOVA, *F*(5, 293) = 0.25; *p* > 0.05 n.s.). By contrast, PC2, reflecting the synergistic accumulation of Cl, K, Ca, and Ba, showed pronounced seasonal variation (Fig. 4), with accumulation peaking in August and declining towards October. Post hoc comparisons using Tukey’s HSD test indicated that October differed significantly from August (*p* < 0.001) and from September (*p *< 0.05), whereas no other pairwise seasonal differences were significant. Seasonal variation had no significant effect on the antagonistic interaction between Mn and Sr (PC3; one-way ANOVA, *F*(5, 293) = 0.97; *p* > 0.05), the synergistic accumulation of Zn and Pb (PC4; one-way ANOVA, *F*(5, 293) = 1.32; *p* > 0.05 n.s.) or on Ca (PC5; Welch *F *= 1.45, df = 133.4, *p* > 0.05 n.s.).

#### Organ type and surrounding soil

**Fig. 5:**
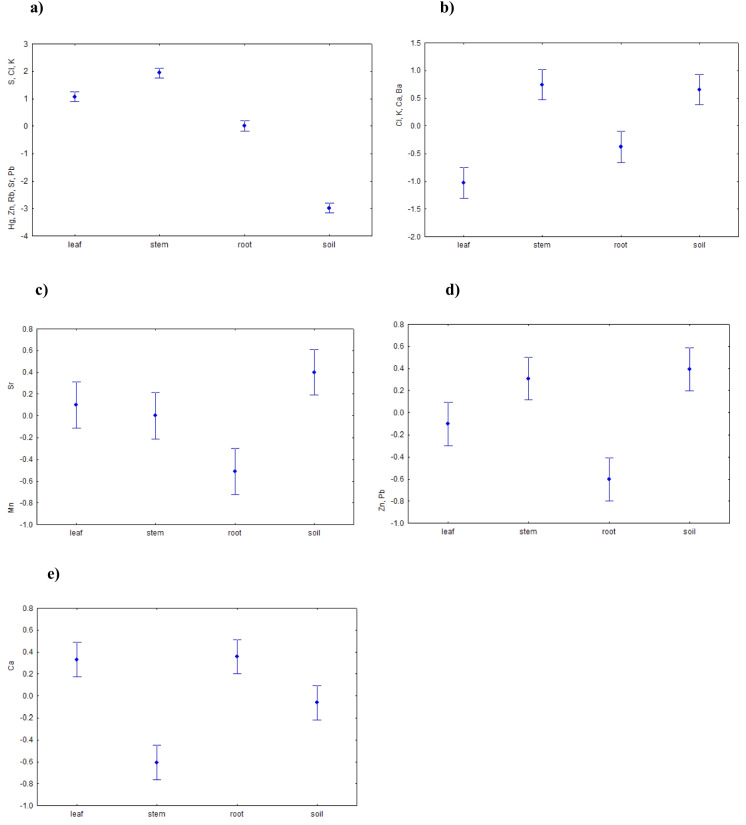
Means (± SE) of PCA scores in relation to plant organ type and surrounding soil: **a** PC1 (38.65% of explained variance) (Welch *F* = 439, df = 189.2, *p* < 0.001); **b** PC2 (20.74%) (Welch *F* = 47, df = 189.6, *p* < 0.001); **c** PC3 (10.13%) (Welch *F* = 12.76, df = 187.5, *p *< 0.001); **d** PC4 (9.16%) (Welch *F* = 41.02, df = 183.3, *p* < 0.001); and **e** PC5 (6.36%) (Welch *F* = 40.91, df = 190.8, *p* < 0.001)

PC1 differed significantly among sample types (plant organ vs. soil; Fig. 5a). According to Tukey’s pairwise test, all comparisons were significant (*p* < 0.001). This axis reflected the antagonistic relationship between biogenic elements (S, Cl, K) and potentially toxic trace elements (Hg, Zn, Rb, Sr, and Pb), depending on organ type. *S. carpatica* accumulated a higher proportion of contaminants in the root relative to the soil, whereas the stem and leaf preferentially accumulated biogenic elements. PC2 was likewise associated with sample type (Fig. 5b), with elevated Cl, K, Ca, and Ba in soil and stem, and the lowest levels in the leaf. PC3 (Fig. 5c) highlighted contrasting behaviour of Mn and Sr: when Sr increased in soil, stem, and leaf, Mn rose in the root, and vice versa. PC4 (Fig. 5d) indicated that Zn and Pb were accumulated mainly in the root and leaf, with lower concentrations in stem and soil. Finally, PC5 (Fig. 5e) captured significant variation in Ca, with higher levels in leaf and root, lower in stem, and intermediate in soil. Significant pairwise differences among sample types for the first five PCs are summarized in Table 6.

**Table 6:** Results of Tukey’s HSD test for differences among plant organs and soil in factor scores (Factors 1–5). Different letters indicate statistically significant differences (*p* < 0.05)

Organ	Factor 1	Factor 2	Factor 3	Factor 4	Factor 5
Leaf	a	a	ab	b	a
Stem	b	c	b	a	b
Root	c	b	c	c	a
Soil	d	c	ab	a	c

Organs sharing the same letter within a factor are not significantly different from each other. Different letters indicate significant differences. In some cases, organs may be assigned to two groups simultaneously (e.g., “ab”), meaning that they do not differ significantly from either group

## Discussion

### Organ-specific partitioning of biogenic vs. potentially toxic trace elements (PC1)

Principal Component 1 (PC1) was not related to changes in site (altitude) (Fig. 3a) or seasonality (Fig. 4a). This pattern indicates that PC1 primarily reflects internal plant partitioning of elements among organs rather than environmental variation along the altitudinal gradient. The lack of a clear altitudinal signal may partly reflect the heterogeneous microhabitat structure of the sampling plots, as some sites were located under spruce canopy whereas others were in open areas. Canopy structure is known to modify element inputs and accumulation patterns, and concentrations of trace elements often increase with elevation in mountain environments, although this relationship is strongly site-specific (Dash et al., 2023; Tužinský & Chudíková, 1991).

In contrast, PC1 showed a strong relationship with sample type (root, stem, leaf, soil; Fig. 5a). Biogenic elements (S, Cl, and K) were most abundant in aboveground tissues, particularly in stems and leaves, whereas roots and surrounding soils were relatively enriched in potentially toxic trace elements (Hg, Zn, Rb, Sr, and Pb). In the Tatra Mountains, elevated concentrations of trace metals in soils and vegetation have often been interpreted as reflecting long-range atmospheric transport from historical and ongoing mining and smelting activities in Slovakia and major industrial centres in the Ostrava–Kraków–Upper Silesia region (Ballová & Janiga, 2018; Ballová et al., 2019; Kompiš & Ballová, 2021; Šoltés et al., 2014). Although direct deposition measurements were not available in this study, the observed spatial patterns are consistent with such atmospheric inputs. Previous studies from the Tatra Mountains indicate that the occurrence of potentially toxic elements (PTEs), particularly Pb, Zn, and Cd, reflects a combination of geogenic background and anthropogenic inputs (Kobza, 2003; Korzeniowska & Krąż, 2020). Elevated concentrations in surface soil horizons, together with decreasing concentrations with depth, suggest that at least part of the contamination originates from external sources, including long-range atmospheric transport (Kobza, 2003). This interpretation is also supported by regional studies from the Tatra Mountains and adjacent areas, which have reported elevated concentrations of PTEs in soils, mosses and lichens, reflecting the combined influence of local anthropogenic sources (e.g., tourism infrastructure) and inputs from industrial regions beyond the study area (Demková et al., 2023; Korzeniowska & Krąż, 2020).

Biogenic elements are taken up primarily from the soil via roots but are efficiently translocated to aboveground organs. This is reflected in the BCF values (Tab. 3), which indicates higher accumulation of K, Cl, S and Ca in stems and leaves than in roots, with the highest BCF observed for Cl in stems. Translocation factors further showed that Cl, K, and S are efficiently transported from roots to stems, whereas transfer to leaves is more limited. In contrast, roots generally act as a physiological barrier for trace metals, resulting in lower concentrations in stems and leaves than in belowground tissues, a pattern widely reported for terrestrial plants (Asati et al., 2016; Phuong et al., 2023; Tong et al., 2022; Yabanli et al., 2014). Contaminant uptake by plants is closely linked to species-specific traits and may also vary substantially among individuals within the same species or cultivar (Faisal et al., 2020). In an experiment with different vegetable species grown in soils contaminated with Cd, Cu, Pb, and Zn, Alexander et al. (2006) showed that not only different species, but even individuals of the same species, differed markedly in contaminant uptake and accumulation. Although these findings are based on model crop species, they support the interpretation that element accumulation patterns observed in wild alpine plants such as *S. carpatica* may likewise be influenced by species-specific physiological traits and intraspecific variability in uptake and internal transport mechanisms.

The tendency for Mn, Zn, Hg, Sr, Ba, and Pb to be preferentially retained in roots (BCF < 1 for most of these elements, except Zn; Table 3) suggests limited upward mobility within the plant and effective root-level retention. Roots are known to protect plants from metal-induced stress through multiple physiological and biochemical mechanisms, including sequestration and immobilization in cell walls and vacuoles (Cuypers et al., 2002). The observed root retention pattern is also consistent with the presence of arbuscular mycorrhizal fungi (AMF), which are known to contribute to plant tolerance to metal stress (Begum et al., 2019). AMF have previously been documented in *S. carpatica* by Zubek et al. (2009), who reported a predominance of coarse AMF compared to fine AMF endophytes (*Glomus tenue*), with shifts in fungal morphology along the altitudinal gradient. Arbuscular mycorrhizal fungi are widespread symbionts of terrestrial plants and play a key role in nutrient and water acquisition (Miransari, 2017). Under metal stress, AMF can contribute to metal immobilization and reduced bioavailability through the production of metal-binding compounds such as glomalins and metallothioneins (Bano & Ashfaq, 2013), thereby enhancing plant tolerance (Miransari, 2011; Nasiri et al., 2022).

Although AMF colonization was not quantified in this study, the combination of low BCF values for most trace elements in aboveground tissues and the pronounced retention of these elements in roots is consistent with a potential buffering role of the root–mycorrhizal complex. At the same time, the higher accumulation of biogenic elements in stems and leaves (Fig. 5a) reflects efficient nutrient translocation, which may be facilitated by mycorrhizal symbiosis, but also by intrinsic physiological traits of *S. carpatica*. The majority of trace metals are known to be stored and metabolized within arbuscules, vesicles and hyphae of AMF within root tissues (Bano & Ashfaq, 2013), which may provide a plausible mechanistic explanation for the organ-specific partitioning captured by PC1.

### Altitudinal and seasonal patterns of biogenic elements (Cl, K, and Ca) and Ba (PC2)

PC2 showed clear altitudinal effects, with concentrations of Cl, K, Ca, and Ba increasing from the lowest site (1137 m a.s.l.) to the highest site (1861 m a.s.l.), while intermediate elevations did not exhibit a consistent monotonic pattern (Fig. 3b). Such elevation-related enrichment is consistent with several interacting environmental and physiological processes. Higher elevation sites generally receive enhanced atmospheric inputs via precipitation. This process can transport both nutrients and trace elements to soils and vegetation (Barbaro et al., 2024). In addition, soil mineral composition and weathering processes at higher elevations may locally increase the availability of Ca and associated cations. Physiological factors may also contribute, as reduced biomass and smaller leaf area of alpine plants can produce a concentration effect, leading to higher elemental concentrations per unit tissue (Grime, 2001; Körner, 2021). Cooler temperatures and higher air humidity at higher elevations can further modulate nutrient cycling and plant uptake dynamics (He et al., 2016). Long-range atmospheric transport of elements such as Ba and Cl from industrial regions may also contribute to the observed enrichment patterns at higher elevations, which often act as effective “collection points” for airborne material (Ballová & Janiga, 2018).

PC2 was also significantly related to seasonality (Fig. 4) and sample type (plant organ vs. soil; Fig. 5b), explaining 20.74% of the total variance. The synergistic co-variation of Cl, K, Ca, and Ba peaked during late summer (August–September) and declined towards autumn (October). This pattern is consistent with enhanced wet deposition during periods of higher precipitation, which was previously observed in the needles of *Abies alba* in relation to paper mill emissions (Grešíková & Janiga, 2017). Importantly, precipitation data used for interpretation were obtained from our own local precipitation monitoring in Javorová dolina during the study period (October 2021–September 2023), which showed maximum precipitation totals between July and September, followed by a decline towards autumn. The seasonal course of PC2 therefore closely follows the seasonal pattern of rainfall-driven atmospheric inputs. While these patterns are consistent with atmospheric inputs, the lack of direct deposition measurements does not allow the relative contribution of atmospheric versus soil-derived sources to be quantified precisely. Because *S. carpatica* frequently occurs in alpine snowbed habitats, prolonged snow cover may influence early-season nutrient availability through snowmelt-derived solute pulses (Costa et al., 2020). However, the late-summer maxima observed in PC2 suggest that the seasonal patterns detected in this study are more likely linked to rainfall-driven deposition and plant physiological dynamics during the peak growing period rather than to snowmelt inputs.

The co-variation of Cl, K, Ca, and Ba observed in PC2 may partly reflect shared atmospheric inputs and deposition processes. In the heavily industrialized area of Zabrze (Upper Silesia, southern Poland), one of the most industrialized regions in Central Europe, historically dominated by coal mining, steel production and nonferrous metal smelting, PM-bound S, Cl, K, Ca, and Fe exhibited among the highest ambient concentrations, reflecting emissions from fuel combustion in stationary sources and road traffic (Rogula-Kozłowska et al., 2015). The strong co-variation of Cl and K is consistent with a shared origin, as these elements are co-emitted during biomass and fossil fuel combustion in the form of fine aerosols (e.g., KCl), which are easily transported over long distances. Zabrze is located northwest of the Tatra Mountains, and under the prevailing north-westerly circulation in Central Europe (Konček et al., 1973), aerosols originating from this region may also be transported towards the Tatras and could be preferentially intercepted at higher elevations. Similar links between precipitation chemistry, canopy processes, and nutrient inputs have been reported from forest ecosystems, where rainfall and throughfall strongly control Ca, S, and K fluxes to soils and vegetation (Liu et al., 2002; Pauliquevis et al., 2012; Pham et al., 2023; Schreck et al., 2012). Given that the lower-elevation sites in Javorová dolina are located within or at the margins of spruce forest, interactions between precipitation, canopy filtering and throughfall may further modulate the seasonal and altitudinal patterns observed along PC2.

The relationship between PC2 and sample type indicates that the synergistic signal of S, Cl, K, Ca, and Ba was most pronounced in stems and soil, and lowest in leaves and roots (Fig. 5b). This pattern suggests that these elements largely enter the plant–soil system via soil solution and are subsequently redistributed within the plant. During periods of higher rainfall, stemflow and throughfall can concentrate solutes around the root zone, enhancing root uptake and subsequent allocation to the stem (Levia & Germer, 2015; Tužinský & Chudíková, 1991). Essential macronutrients such as S, Cl, K, and Ca play central roles in plant metabolism and structure (Marschner, 2012). In our dataset, BCF values for K, S, Cl, and Ca were highest for stems, consistent with the physiological role of K in energy metabolism and translocation processes (Phuong et al., 2023; Sardans & Peñuelas, 2021), and with the high mobility of Cl⁻ within plant tissues (Colmenero-Flores et al., 2019; White & Broadley, 2001). In alpine plants such as *S. carpatica*, reduced leaf area at higher elevations may further favor the relative accumulation of mobile ions in stems (Kiełtyk, 2024).

Barium behaved synergistically with these biogenic elements despite being potentially phytotoxic at elevated concentrations (de Souza Cardoso et al., 2023). Ba is associated with traffic-related emissions and fossil fuel combustion (Monaci & Bargagli, 1997; Navarro-Ciurana et al., 2023). The synergistic behavior of Ba with Ca is further supported by their chemical similarity as alkaline earth metals; Ba ions can utilize the same transmembrane transport systems and calcium channels, leading to coupled uptake and translocation from the soil solution to the stems. Its co-variation with Cl, K, and Ca is suggestive of a partially shared atmospheric source, a hypothesis that warrants further investigation through direct deposition monitoring.. In addition to mineral weathering, nutrient cations available to plants are supplied by atmospheric deposition and stored in soils as exchangeable cations on the cation-exchange complex (Bel et al., 2020), which may further contribute to the coupled behaviour of these elements captured by PC2.

### Canopy-mediated Mn–Sr antagonism and geomorphological controls (PC3)

Principal component 3 (PC3) captured a pronounced antagonistic relationship between Mn and Sr across sites (Fig. 3c). Higher Mn and lower Sr concentrations characterized sites located under closed spruce canopy (sites 2–4; 1213–1391 m a.s.l.), whereas open sites at the forest edge, upper forest limit, subalpine and alpine belt (sites 1, 5–7) showed the opposite pattern, with relatively higher Sr and lower Mn. This spatial contrast highlights the strong role of canopy structure in modulating element inputs and redistribution.

Spruce crowns intercept aerosols and modify throughfall chemistry, which promotes Mn enrichment in understory soils and vegetation, particularly during wet periods (Małek & Astel, 2008; Nihlgård, 1970). In contrast, open and canopy-gap sites are more directly exposed to wind-driven deposition and resuspension of mineral dust, favoring the accumulation of lithogenic trace elements such as Sr, especially via dry and snow-season deposition (Tan et al., 2019). Seasonal changes in canopy filtration efficiency may further modulate these processes, with enhanced washout during wet periods and stronger interception during drier or colder conditions (Tan et al., 2019). In our dataset, however, PC3 did not show a significant seasonal effect (KW-H (5; 300) = 4.90; *p* > 0.05), suggesting that the Mn–Sr contrast primarily reflects spatial controls rather than short-term seasonal dynamics.

Local geomorphology and soil properties further shaped the PC3 pattern. Site 4, situated on coarse deluvial sediments with a sandy–gravelly texture, exhibited reduced Sr but elevated Mn concentrations. While low cation-retention capacity of coarse substrates promotes Sr leaching (Dube et al., 2022; Fest et al., 2005), Mn enrichment at this site is more plausibly linked to redox- and pH-driven processes. Under acidic conditions, Mn availability increases through desorption into soil solution and through reductive dissolution of Mn oxides under periodically reducing conditions, releasing soluble Mn2⁺ (Alejandro et al., 2020; Kabata-Pendias, 2010). Periodic water saturation in deluvial sediments may therefore enhance Mn mobilization and retention in exchangeable soil fractions, whereas Sr remains more prone to leaching. Similar patterns of Mn enrichment in forest soils and vegetation have been reported across temperate forest ecosystems, where foliar Mn concentrations closely reflected Mn availability in forest floor and mineral soil horizons, highlighting the importance of internal ecosystem cycling processes, including uptake, throughfall and litterfall, and soil chemical conditions in regulating Mn distribution (Richardson, 2017).

The Mn–Sr antagonism observed in PC3 likely reflects contrasting biogeochemical pathways rather than direct ionic competition. Mn mobility is enhanced in acidic and reducing microsites rich in organic matter (Alejandro et al., 2020; Marschner, 2012), whereas Sr uptake may also be linked to atmospheric inputs (Burger & Lichtscheidl, 2019) and often follows Ca dynamics in plants, although Ca generally dominates uptake in acidic forest soils (Poszwa et al., 2000). Both elements behave as alkaline-earth cations with partially overlapping transport pathways in soils and plants, and Sr may substitute for Ca in plant tissues (Alejandro et al., 2020; Kabata-Pendias, 2010).

Organ-specific patterns further support these contrasting controls: Mn tended to be relatively enriched in roots under canopy-influenced, acidic or periodically reducing conditions, while Sr showed higher relative representation in foliage and surface soil at open, deposition-exposed sites (Figs.). Overall, PC3 thus reflects a spatially structured interplay between canopy-mediated modification of deposition and throughfall chemistry, geomorphological controls on soil redox and texture, and the differing biogeochemical behaviour of Mn and Sr.

### Co-occurrence of Zn and Pb and elevation-related deposition patterns (PC4)

Principal component 4 (PC4) captured the coupled behaviour of Zn and Pb, with significantly higher scores at the highest open alpine site compared to forested localities (Fig. 3d). This altitudinal pattern is consistent with previous findings from the Tatra Mountains, where the alpine vegetation belt exhibited substantially higher Zn and Pb loads than forest zones (Ballová et al., 2020). Similar co-variation of Zn and Pb has been reported from the Gorce Mountains in southern Poland, north of the Tatras, where topsoil concentrations of Pb and Zn were strongly correlated, reflecting common source regions linked to metal smelting, coal mining and steel production in the Silesia–Kraków industrial area (Miśkowiec, 2022). The Gorce massif acts as a partial orographic barrier for aerosols transported from these regions; however, due to its lower elevation relative to the Tatras, a substantial fraction of airborne particles may continue southwards and be preferentially intercepted by the higher Tatra ridges, potentially forming an efficient secondary accumulation zone for atmospherically transported Zn and Pb. Similar patterns have been documented in soils and vegetation across the Tatra National Park, where elevated concentrations of Pb, Cd, and Zn have been linked to both long-range transport from industrial regions and local anthropogenic influences, although the relative contribution of these sources varies among sites (Ciarkowska & Miechówka, 2022; Korzeniowska et al., 2021).

Seasonal variation in PC4 scores was not statistically significant, indicating that Zn and Pb inputs were relatively stable over the sampled months and likely influenced by long-range atmospheric transport rather than short-term local variability (Harmens et al., 2010). The strong co-occurrence of Zn and Pb in plant organs, with the highest concentrations in roots and the lowest in stems and surrounding soil (Fig. 5d), suggests efficient retention of these metals within the rhizosphere. This pattern is consistent with the high binding affinity of Pb2⁺ and Zn2⁺ to root cell walls and exudates, which limits their mobility in the xylem and restricts translocation to aboveground tissues (Kabata-Pendias, 2010; Manara, 2012; Ricachenevsky et al., 2018).

Overall, PC4 highlights the combined influence of elevation-related exposure, reduced canopy filtering at open alpine sites, and plant-internal retention mechanisms on the spatial distribution of Zn and Pb. In this context, *S. carpatica* appears to reflect the integrated signal of atmospherically supplied trace metals under contrasting canopy and altitudinal conditions, rather than direct local emission sources.

### Calcium variability across sites and organ-specific allocation (PC5)

Principal component 5 (PC5) primarily captured variability in Ca concentrations among sites and sample types. Although altitude had a statistically significant effect on Ca represented by PC5, this relationship did not follow a monotonic altitudinal gradient (Fig. 3e). Calcium concentrations varied among sites, with a pronounced minimum at site 4 (1391 m a.s.l.). In contrast to some previous studies conducted under spruce canopies, where throughfall increased Ca inputs relative to open sites due to dry deposition and canopy leaching (Hansen, 1996; Nihlgård, 1970; Novo et al., 1992), the lowest Ca levels in our study occurred at a forested site. This discrepancy likely reflects the strong control of local soil properties on Ca availability. Calcium availability is closely linked to soil pH and texture, with deficiencies commonly occurring in acidic, coarse-textured soils (Borges et al., 2012). This may explain the low Ca values at site 4, which was characterized by sandy–gravelly deluvial sediments despite being located beneath spruce canopy. In addition, isotopic evidence indicates that a substantial proportion of soil Ca can originate from atmospheric sources (31–98%; Bélanger & Holmden, 2010), suggesting that Ca availability in montane–alpine systems may reflect a balance between atmospheric inputs, mineral weathering and site-specific soil conditions.

Across plant organs and soil, mean Ca concentrations were generally highest in leaves and stems, followed by roots and soil (Tables 1 and 2). However, PC5 revealed that this pattern was not universal: under certain environmental conditions, stems exhibited markedly lower Ca concentrations than roots and leaves, while soil showed intermediate values (Fig. 5e), providing a more context-specific perspective on organ-level Ca partitioning relative to the overall patterns shown in Fig. 2b. This deviation likely reflects the combined influence of soil chemistry and plant-internal allocation strategies. Soil acidification reduces the pool of exchangeable Ca, promotes leaching, and increases competition with H⁺ and Al3⁺ ions, thereby constraining Ca supply to plant tissues (Hue, 2022; Jiang et al., 2018). Acid deposition and canopy-mediated throughfall can further lower available Ca in forest soils (Bredemeier, 1988; Likens et al., 1996). Under such Ca-limited conditions, plants may preferentially allocate Ca to organs essential for physiological stability and function, particularly roots (membrane integrity and ion exchange; Wdowiak et al., 2024) and leaves (cell wall strengthening and stomatal regulation; White & Broadley, 2003), at the expense of stems.

As a calcifuge species with low root cation exchange capacity and a high shoot K/Ca ratio (Broadley et al., 2003; Kinzel & Lechner, 1992), *S. carpatica* is likely adapted to low-Ca environments and may exhibit flexible Ca partitioning among organs depending on local edaphic constraints. Overall, PC5 highlights that Ca distribution in *S. carpatica* reflects the interplay between site-specific soil properties, atmospheric inputs, and physiological allocation priorities, rather than a simple altitudinal trend.

### *S. carpatica* in the context of alpine bioindicators

In alpine and montane ecosystems, biomonitoring of atmospheric pollutants has traditionally relied on organisms such as lichens, mosses, and ericaceous shrubs (e.g., *Vaccinium* spp.). Lichens and mosses are particularly effective indicators of atmospheric deposition in remote mountain areas because they lack a protective cuticle and absorb elements directly from wet and dry deposition, allowing them to accumulate metals in higher concentrations than most vascular plants (Brunialti & Frati, 2023; Budzyńska-Lipka et al., 2022). However, these organisms mainly reflect atmospheric inputs and provide limited information on soil–plant interactions.

Vascular plants such as *Vaccinium myrtillus* integrate both atmospheric deposition and soil-derived element uptake, although element accumulation in leaves may reflect only a relatively short seasonal absorption period during the growing season (Korzeniowska et al., 2025). Recent research further demonstrated that elemental composition in perennial stem tissues of *V. myrtillus* is strongly influenced by elevation-driven environmental gradients, while plant age contributes to long-term accumulation patterns, supporting its use as a bioindicator of environmental change in high-mountain ecosystems (Ballová et al., 2026).

*Soldanella* species represent a different ecological strategy typical of alpine snowbed habitats. They are capable of developing under deep snow cover (up to 2–3 m) and follow an internal phenological clock that enables rapid growth immediately after snowmelt. Although individual plants are relatively short-lived compared to woody shrubs, they form clonal colonies that may persist for long periods in stable alpine environments (Körner et al., 2019). In addition, *Soldanella* produces overwintering basal leaves that persist beneath the snowpack and resume physiological activity soon after snowmelt. This phenological strategy may increase their sensitivity to element inputs associated with snow accumulation and meltwater pulses, which represent an important pathway of atmospheric deposition in alpine environments (Avak et al., 2019).

In this context, *S. carpatica* may provide complementary insights for alpine biomonitoring, as the analysis of different plant organs enables simultaneous assessment of element uptake from soils and internal redistribution within plant tissues, offering a more integrated perspective on element accumulation processes in montane–alpine ecosystems.

## Conclusions

This study provides new insights into the elemental composition of *S. carpatica* along a montane–alpine gradient in the Tatra Mountains. The transect was deliberately designed to capture environmental conditions characteristic of many Tatra valleys, encompassing forested, subalpine, and alpine zones, and may therefore be regarded as a representative model of montane–alpine gradients within the range. In this context, the observed patterns likely reflect processes operating more broadly across the Tatra Mountains. Nevertheless, extrapolation to other mountain systems or generalization of element accumulation patterns in *S. carpatica* throughout its entire geographic distribution should be approached with caution, as geological substrate, climatic regimes, and atmospheric deposition histories may differ among regions.

Seasonal analyses were restricted to the main growing period, as samples from December to May were excluded due to persistent snow cover and insufficient material. Although sampling extended over two consecutive years, the short alpine vegetation season limited the scope for detailed interannual comparisons. In addition, microhabitat variability associated with alpine snowbed dynamics was not explicitly quantified. Because *S. carpatica* commonly occurs in snowbed habitats where prolonged snow cover may influence nutrient inputs and element accumulation, this factor may contribute to local variability along the gradient.

Elemental concentrations were determined using portable ED-XRF, representing a screening-level, semi-quantitative analytical approach. Calibration with certified reference materials enhanced reliability; however, matrix effects and detection constraints, particularly for lighter elements, may introduce minor uncertainty in absolute concentrations. Consequently, interpretation focused primarily on relative patterns and multivariate relationships rather than absolute thresholds.

The results demonstrate that the elemental composition of *S. carpatica* reflects the combined influence of canopy structure, altitude, precipitation dynamics, and soil properties. Biogenic elements (S, Cl, K, and Ca) were preferentially accumulated in aboveground tissues and exhibited efficient translocation, whereas most potentially toxic trace elements (Hg, Pb, Sr, and Ba; partly Zn) were retained predominantly in roots, suggesting limited upward mobility and effective buffering within the root–soil interface.

Altitudinal effects were most evident for Cl, K, Ca, and Ba (PC2), which increased towards higher elevations, consistent with potentially enhanced atmospheric inputs and reduced canopy interception at open sites. The Mn–Sr antagonism (PC3) reflected canopy-mediated throughfall and geomorphological controls, whereas the coupled behaviour of Zn and Pb (PC4) is consistent with a potential influence of long-range atmospheric transport. Calcium variability (PC5) did not follow a simple elevational trend but instead reflected the interaction among soil chemistry, substrate texture, possible atmospheric contributions, and organ-specific allocation strategies.

Overall, these findings indicate that *S. carpatica* integrates signals arising from both atmospheric deposition and local edaphic conditions, supporting its potential relevance for high-mountain environmental monitoring. Future research could extend this approach to additional valleys and incorporate direct deposition measurements and complementary laboratory analyses to improve source attribution and ecological interpretation.

## Supplementary Information

Below is the link to the electronic supplementary material.

ESM 1 (DOCX 31.1 KB)

## Data Availability

The datasets presented in this article are not readily available because they are owned by the University of Žilina and are subject to institutional licensing and intellectual property regulations. Data may be provided by the authors upon reasonable request. Requests should be directed to the corresponding author.

## Declarations

“All authors have read, understood, and have complied as applicable with the statement on “Ethical responsibilities of Authors” as found in the Instructions for Authors”.
