# Supplementary material for: Element accumulation patterns and bioindicator potential of Soldanella carpatica along a montane–alpine gradient
Source: Environ Monit Assess. 2026 May 22;198(6):631. doi: 10.1007/s10661-026-15471-2 (PMC13197334; doi:10.1007/s10661-026-15471-2)
Supplement: Supplementary file 1 — (DOCX 31.1 KB) [file 10661_2026_15471_MOESM1_ESM.docx]

**Supplementary Information** to the paper

**Element accumulation patterns and bioindicator potential of *Soldanella carpatica* along a montane–alpine gradient**. *Environmental Monitoring and Assessment*

Zuzana Kompišová Ballová^1^*, Gabriela Zatkalíková^1^, Marián Janiga^1^

^1^Institute of High Mountain Biology, Žilina University, Tatranská Javorina 7, 05956 Tatranská Javorina, Slovak Republic, author's email address: ballova1@uniza.sk, gzatkalikova@gmail.com, janiga@uniza.sk

*Corresponding author: ballova1@uniza.sk, tel./fax.: +421 52 449 9108, ORCID 0000-0002-4518-2763

**Supplementary Table S1** Sampling design of *Soldanella carpatica* (October 2021–September 2023): number of samples collected per organ across altitude levels and sampling periods

| **Altitude [m a.s.l.]** | **Period** | **Leaf (N)** | **Stalk (N)** | **Root (N)** | **Soil (N)** | **Total (N)** |
| --- | --- | --- | --- | --- | --- | --- |
| 1137 | May | 2 | 2 | 2 | 2 | 8 |
|  | June | 2 | 2 | 2 | 2 | 8 |
|  | July | 2 | 2 | 2 | 2 | 8 |
|  | August | 2 | 2 | 2 | 2 | 8 |
|  | September | 2 | 2 | 2 | 2 | 8 |
|  | October | 2 | 2 | 2 | 2 | 8 |
|  | November | 2 | 2 | 2 | 2 | 8 |
|  | December–April | 4 | 4 | 3 | 4 | 15 |
| 1213 | May | 2 | 2 | 2 | 2 | 8 |
|  | June | 2 | 2 | 2 | 2 | 8 |
|  | July | 2 | 2 | 2 | 2 | 8 |
|  | August | 2 | 2 | 2 | 2 | 8 |
|  | September | 2 | 2 | 2 | 2 | 8 |
|  | October | 2 | 2 | 2 | 2 | 8 |
|  | November | 2 | 2 | 2 | 2 | 8 |
|  | December–April | 1 | 1 | 1 | 1 | 4 |
| 1259 | May | 2 | 2 | 2 | 2 | 8 |
|  | June | 2 | 2 | 2 | 2 | 8 |
|  | July | 2 | 2 | 2 | 2 | 8 |
|  | August | 2 | 2 | 2 | 2 | 8 |
|  | September | 2 | 2 | 2 | 2 | 8 |
|  | October | 2 | 2 | 2 | 2 | 8 |
|  | November | 2 | 2 | 2 | 2 | 8 |
|  | December–April | 0 | 0 | 0 | 0 | 0 |
| 1391 | May | 2 | 2 | 2 | 2 | 8 |
|  | June | 2 | 2 | 2 | 2 | 8 |
|  | July | 2 | 2 | 2 | 2 | 8 |
|  | August | 1 | 2 | 2 | 2 | 7 |
|  | September | 2 | 2 | 2 | 2 | 8 |
|  | October | 2 | 2 | 2 | 2 | 8 |
|  | November | 1 | 1 | 1 | 0 | 3 |
|  | December–April | 0 | 0 | 0 | 0 | 0 |
| 1473 | May | 0 | 0 | 0 | 0 | 0 |
|  | June | 2 | 2 | 2 | 2 | 8 |
|  | July | 2 | 2 | 2 | 2 | 8 |
|  | August | 2 | 2 | 2 | 2 | 8 |
|  | September | 2 | 2 | 2 | 2 | 8 |
|  | October | 2 | 2 | 2 | 2 | 8 |
|  | November | 1 | 1 | 1 | 1 | 4 |
|  | December–April | 0 | 0 | 0 | 0 | 0 |
| 1554 | May | 0 | 0 | 0 | 0 | 0 |
|  | June | 2 | 2 | 2 | 2 | 8 |
|  | July | 2 | 2 | 2 | 2 | 8 |
|  | August | 2 | 2 | 2 | 2 | 8 |
|  | September | 2 | 2 | 1 | 2 | 7 |
|  | October | 2 | 2 | 2 | 2 | 8 |
|  | November | 1 | 1 | 0 | 1 | 3 |
|  | December–April | 0 | 0 | 0 | 0 | 0 |
| 1861 | May | 0 | 0 | 0 | 0 | 0 |
|  | June | 2 | 2 | 2 | 2 | 8 |
|  | July | 1 | 1 | 1 | 1 | 4 |
|  | August | 2 | 1 | 2 | 2 | 7 |
|  | September | 1 | 1 | 1 | 1 | 4 |
|  | October | 1 | 1 | 1 | 1 | 4 |
|  | November | 1 | 1 | 1 | 1 | 4 |
|  | December–April | 0 | 0 | 0 | 0 | 0 |

**Supplementary Table S2** Number of samples of *Soldanella carpatica* used in seasonal analyses, aggregated by sampling month (June–November) across all altitude levels and organs

| **Month** | **Leaf (N)** | **Stalk (N)** | **Root (N)** | **Soil (N)** | **Total (N)** |
| --- | --- | --- | --- | --- | --- |
| June | 14 | 14 | 14 | 14 | 56 |
| July | 13 | 13 | 13 | 13 | 52 |
| August | 13 | 13 | 14 | 14 | 54 |
| September | 13 | 13 | 12 | 13 | 51 |
| October | 13 | 13 | 13 | 13 | 52 |
| November | 10 | 9 | 9 | 9 | 38 |

**Foodnote:** Only samples from June to November were included in seasonal analyses due to limited accessibility and insufficient sample availability during winter and early spring (December–May).

**Supplementary Table S3** Accuracy verification of ED-XRF measurements using certified reference materials (CRMs) for plant and soil matrices, including certified concentrations, calibrated mean values, percentage recovery, and relative standard deviation (RSD) for the elements evaluated in this study

| **CRM** | **Element** | **Certified (mg/kg)** | **Mean_calibrated (mg/kg)** | **Recovery (%)** | **RSD (%)** |
| --- | --- | --- | --- | --- | --- |
| INCT-PVTL-6 Tobacco Leaves | Ca | 22,970 | 22,840 | 99.4 | 2.8 |
|  | K | 26,400 | 26,130 | 99.0 | 2.5 |
|  | S | 3,780 | 3,690 | 97.6 | 3.1 |
|  | Mn | 136 | 132 | 97.1 | 4.2 |
|  | Rb | 5.97 | 6.10 | 102.2 | 5.3 |
|  | Sr | 133 | 129 | 97.0 | 3.6 |
|  | Zn | 43.6 | 44.2 | 101.4 | 4.8 |
|  | Ba | 41.6 | 38.9 | 93.5 | 6.2 |
|  | Pb | 0.972 | 0.95 | 97.7 | 7.4 |
| SRM 1575a Pine Needles | K | 4,170 | 4,010 | 96.1 | <1 |
|  | Ca | 2,500 | 2,350 | 94.0 | <1 |
|  | Cl* | 421 | 347 | 82.3 | 15.9 |
|  | Rb | 16.5 | 17.7 | 107.1 | 1.4 |
|  | Zn | 38 | 39.7 | 104.4 | 6.3 |
|  | Ba* | 6.0 | 4.2 | 70.6 | 13.4 |
| SRM 2711a Montana II Soil | Mn | 675 | 672 | 99.6 | 1.9 |
|  | Zn | 414 | 412 | 99.4 | 2.3 |
|  | Rb | 120 | 121 | 100.5 | 0.9 |
|  | Sr | 242 | 241 | 99.6 | 0.8 |
|  | Ba | 730 | 729 | 99.8 | 1.1 |
|  | Pb | 1,400 | 1,399 | 100.0 | 0.2 |
| ERM-CC141 Loam Soil | Mn | 464 | 463 | 99.8 | 0.4 |
|  | Zn | 57 | 56 | 98.4 | 1.4 |
|  | Pb | 41 | 42 | 101.9 | 3.2 |

* values marked with an asterisk are informative only (non-certified or outside optimal ED-XRF calibration range)

**Supplementary Table S4** Matrix-dependent limits of detection (LOD) and quantification (LOQ) for ED-XRF analysis (in mg/kg dry wt.) derived from blank measurements of representative plant and soil matrices

| **Element** | **LOD starch** | **LOQ starch** | **LOD SiO₂** | **LOQ SiO₂** | **LOD agarose** | **LOQ agarose** | **LOD paraffin** | **LOQ paraffin** |
| --- | --- | --- | --- | --- | --- | --- | --- | --- |
| S | 104 | 347 | 209 | 698 | 113 | 377 | 108* | 360* |
| Cl | 133* | 444* | 238 | 792 | 110* | 365* | 103* | 343* |
| K | 77* | 258* | 110 | 368 | 45* | 150* | 39* | 130* |
| Ca | 34* | 114* | 51 | 169 | 57* | 190* | 19 | 64 |
| Mn | 4.7* | 15.7* | 8.0 | 26.7 | 4.8* | 16.0* | 5.1* | 17.0* |
| Zn | 5.0 | 16.7 | 8.0 | 26.7 | 5.3 | 17.7 | 5.3 | 17.7 |
| Rb | 1.8* | 6.0* | 2.2 | 7.3 | 2.0* | 6.7* | 2.1* | 7.0* |
| Sr | 1.1 | 3.7 | 2.1* | 7.0* | 1.1 | 3.7 | 1.0 | 3.3 |
| Ba | 13 | 43 | 29 | 97 | 14 | 47 | 15* | 50* |
| Pb | 4.5 | 15 | 6.0 | 20 | 4.7 | 16 | 5.1* | 17.0* |

* elements detected in blanks; LOD estimated from instrumental uncertainty (LOD = 3σ). LOQ values were calculated as LOQ = (10/3) × LOD.
